# Fixed-Rate Universal Lossy Source Coding and Model Identification: Connection with Zero-Rate Density Estimation and the Skeleton Estimator

**DOI:** 10.3390/e20090640

**Published:** 2018-08-25

**Authors:** Jorge F. Silva, Milan S. Derpich

**Affiliations:** 1 Information and Decision System Group, Department of Electrical Engineering, Universidad de Chile, Av. Tupper 2007, Santiago 7591538, Chile; 2 Department of Electronic Engineering, Universidad Tecnica Federico Santa Maria, Valparaiso 2390123, Chile

**Keywords:** fixed-rate lossy source coding, joint coding and modeling, universal source coding, learning with rate constraints, the skeleton estimator, *L*_1_-totally bounded classes

## Abstract

This work demonstrates a formal connection between density estimation with a data-rate constraint and the joint objective of fixed-rate universal lossy source coding and model identification introduced by Raginsky in 2008 (IEEE TIT, 2008, 54, 3059–3077). Using an equivalent learning formulation, we derive a necessary and sufficient condition over the class of densities for the achievability of the joint objective. The learning framework used here is the skeleton estimator, a rate-constrained learning scheme that offers achievable results for the joint coding and modeling problem by optimally adapting its learning parameters to the specific conditions of the problem. The results obtained with the skeleton estimator significantly extend the context where universal lossy source coding and model identification can be achieved, allowing for applications that move from the known case of parametric collection of densities with some smoothness and learnability conditions to the rich family of non-parametric $L_{1}$-totally bounded densities. In addition, in the parametric case we are able to remove one of the assumptions that constrain the applicability of the original result obtaining similar performances in terms of the distortion redundancy and per-letter rate overhead.

## 1. Introduction

Universal source coding (USC) has a long history in information theory and statistics [1,2,3,4,5]. Davisson’s seminal work [4] formalized the variable-length lossless coding problem and introduced important information quantities for performance analysis [1,2]. In this lossless setting, it is well-understood that the Shannon entropy provides the minimum achievable rate (in bits per sample) [2] to code a stationary and memoryless source when the probability (model) of the source is available. When the probability of the source is not known but belongs to a family of distributions F (the so called universal source coding problem), the focus of the problem is to characterize the penalty (or redundancy in bits per sample) that an encoder and decoder pair will experience due to the lack of knowledge about the samples’ probability [1]. In the lossless case, a seminal result states that the least worst-case redundancy over F (or the minimax solution of the USC problem for F) is determined by the information radius of F [1].

Building on this connection between least worse-case redundancy and information radius of F, there are numerous important results developed for lossless USC [1,6,7,8,9]. In particular, it is known that the information radius grows sub-linearly (with the block-length) for the family of finite alphabet stationary and memoryless sources [1], which implies the existence of a universal source code that achieves Shannon entropy as the block length goes to a large value for every distribution in F. However universality is not possible for the family of alphabet stationary and memoryless sources because the information radius of this family is unbounded [3,5,7]. More recent results on lossless USC over countable infinite alphabets have looked at restricting the analysis to specific collections of distributions (with some tail bounded conditions) to achieve minimax universality [7,8,9] and also looked at weak variations of the lossless source coding setting [10,11,12].

In the fixed-rate lossy source coding problem, assuming first that the probability μ of a memoryless source is known, the performance limit of the coding problem is given by the Shannon distortion-rate function $D_{\mu }\left(R\right)$ [2,13]. Consequently, the universal lossy source coding problem reduces to compare the distortion of a coding scheme (satisfying a fixed-rate constraint) with the Shannon distortion-rate function assuming that the designer only knows that μ∈F. The literature on this problem is rich [3,5,14,15,16,17,18] with a first result dating back to Ziv [17] who showed the existence of weakly minimax fixed-rate universal lossy source code for the class of stationary sources under certain assumptions about the source, the alphabet, and the distortion measure. More refined results were presented in [5,16] one of which established necessary and sufficient conditions to achieve weakly minimax universality for the class of stationary and ergodic sources. To provide a more specific analysis of universal lossy source coding, Linder et al. [14] presented a lossy USC scheme with a distortion redundancy that goes to zero as $O(\sqrt{\frac{\log\log n}{\log n}})$ for the case of independent and identically distributed (i.i.d.) bound sources. Later Linder et al. [15] improved previous results showing a fixed-rate lossy construction with a distortion redundancy that vanishes as $O(n^{-1}\log n)$ and $O(\sqrt{n^{-1}\log n})$ with *n* for finite alphabet i.i.d. sources and bounded infinite alphabet i.i.d. sources, respectively. Similar convergence results were obtained using a nearest-neighbor vector quantization approach in [19].

It is also understood that universal variable length lossless-source coding is connected with the problem of distribution estimation [3,6,20] as there is a one-to-one correspondence between prefix-free codes and finite-entropy discrete distributions in the finite and countable alphabet case [1,2,21]. Building on this one-to-one correspondence in the lossless case, Györfi et al. ([3], Theorem 1) showed that the redundancy (in bits per sample) of a given code upper bounds the expected divergence between the true distribution of the source μ and the estimated distribution derived from the code. Therefore, the existence of a universal (lossless) source code for F implies the existence of a universal (distribution-free in F) estimator of the distribution in expected (direct) information divergence [22]. This means that achieving lossless USC not only provides a lossless representation of the data, but it offers a consistent (error-free) estimator of the distribution at the receiver.

The connection between coding and distribution estimation that is evident in the lossless case is not, however, present in the (fixed-rate) lossy source coding problem. As argued in [18], a fixed-rate lossy source code does not offer a direct map with a probability distribution (model) for the source. In light of this gap between lossy codes and distributions (models) and motivated by some problems in adaptive control, where it is relevant to both compress data in a lossy way and identify the distribution of the source at the receiver [18,23], Raginsky explored the joint objective of fixed-rate universal lossy source coding and model (i.e., distribution) identification in [18].

Inspired by Rissanen’s achievability construction in [6,20], Raginsky [18] proposed a new setting for the problem of fixed-rate universal lossy compression of continuous memoryless sources based on the idea of a two-stage joint coding and model or distribution identification framework. In this context, he proposed a two-stage scheme to consider two objectives: fixed-rate universal lossy source coding and source distribution (model) identification. The first objective of the scheme is to transmit the data (optimally) in the classical distortion-rate sense [24], while the second objective is to learn and transmit a description (quantized version) of the source distribution (model) [25,26]. Taking ideas from statistical learning, Raginsky proposed [18] splitting the data into training and testing samples. The training data is used in the first-stage of the encoding process to construct a quantized estimation of the source distribution and encode it (the first stage bits). Then in a second stage of the encoding process, the first-stage bits are used to pick a matched (with the estimated distribution) fixed-rate lossy source code to encode the test data (the second stage bits). In this joint coding and modeling setting, the existence of a zero-rate consistent estimator of the density (in expected total variation) is sufficient to show the existence of a weakly minimax universal fixed-rate source coding scheme [18] (Theorem 3.2), achieving the Shannon distortion-rate function [2,24,27,28], for any given rate. This result is obtained for a wide class of single-letter bounded distortion functions and for a family of source densities $F=\left\{\mu_{\theta }:\theta \in \Theta \right\}$ indexed over a bounded finite dimensional space $\Theta \subset {⊗}_{i=1}^{k}\left[-L,L\right]\subset R^{k}$ (i.e., a parametric collection) with some needed smoothness and learnability conditions [18] (Theorem 3.2).

It is important to highlight that the joint coding and modeling achievability results in [18] did not degrade the performance of the source coding objective. In fact by restricting the analysis to the source coding objective alone, the joint coding and modeling framework in [18] showed the same state-of-the-art performance results as conventional two-stage universal source coding schemes (or universal vector quantizers) [14,15,19] in terms of distortion redundancy and per-letter rate overhead ($O(\sqrt{\log(n)/n})$ and O(log(n)/n), respectively) as the block length *n* tends to a large number. Importantly, the first-stage bits of this joint coding and modeling scheme are used to achieve model identification at the receiver with arbitrary precision in total variation (with a rate of convergence of $O(\sqrt{\log(n)/n})$ as *n* goes to infinity), with no extra cost in bits per-letter compared with conventional fixed-rate lossy source coding methods.

### Contributions of This Work

This work formally studies the interplay between density estimation under a data-rate constraint and the joint fixed-rate universal lossy source coding and modeling problem with training data or memory introduced in [18]. The first main result (Theorem 1) establishes a connection between zero-rate density-estimation and a universal joint coding and modeling scheme that achieves optimal lossy source coding (in a distortion-rate sense) and lossless model identification. This result is obtained for the general family of bounded single-letter distortions [13]. Remarkably, this connection implies that the construction of a joint coding and modeling scheme reduces to the construction of a zero-rate density estimator. From this result, the second main result (Theorem 2) stipulates a necessary and sufficient condition for the existence of a weakly minimax universal joint coding and modeling scheme. For the achievability part of this result, we used the skeleton estimator as our learning framework [29]. Using this learning framework we extend the parametric context explored in [18] to the rich non-parametric scenario of $L_{1}$-totally bounded densities [30].

Furthermore, revisiting the parametric case studied in [18], by using the skeleton estimator we are able to remove some of the assumptions that limit the applicability of the original result. We show that the skeleton estimator matches the best performance reported in [18] in terms of the distortion redundancy and (per-letter) rate overhead, in particular obtaining rates of convergence to zero of $O(\sqrt{\log(n)/n})$ and O(log(n)/n), respectively, as the block-length tends to infinity. To obtain this, our result relaxes the finite Vapnik and Chervonenkis (VC) dimension assumption considered in [18]. On the other hand, when the finite VC dimension assumption is added in the analysis, the skeleton learning scheme offers a convergence rate of $O(1/\sqrt{n})$ for the distortion redundancy as the sample-length goes to infinity. Finally, the skeleton framework is implementable in the parametric case as its minimum-distance decision is carried out on a finite number of candidates and the oracle ϵ-skeleton (or the ϵ-covering in total variation of F) [30] (Chapter 7) can be replaced by a practical uniform covering of the compact index set $\Theta \subset R^{k}$ (Theorem 4). Finally, it is worth noting that a preliminary version of this work (in the context of density estimation under a data-rate constraint) was presented in [31].

The rest of the paper is organized as follows: Section 2 introduces the setting of the joint coding and modeling with training data. Section 3 elaborates the connections with zero-rate density estimation. Section 4 presents the main joint coding and modeling result (Theorem 2) and introduces the skeleton estimator. Finally, Section 5 revisits a special case where the distributions are indexed by finite dimensional bounded space (the parametric context). A summary of the results is presented in Section 6 and Section 7. Finally, the proofs are presented in Section 8.

## 2. Preliminaries

The fixed-rate coding and modeling problem introduced in [18] is presented in this section. This joint coding and modeling problem will be the main focus of this work. In addition, notations and definitions used in the rest of the paper will be presented.

### 2.1. Basic Definitions

Let $X\in B(R^{d})$ be a separable and complete subset of $R^{d}$ where $B(R^{d})$ is the Borel sigma field. Let P(X) be the collection of probability measures on (X,B(X)), with B(X) denoting the Borel sigma field restricted to X, and let AC(X)⊂P(X) denote the set of probability measures absolutely continuous with respect to the Lebesgue measure λ [32]. For any μ∈AC(X), $g_{\mu }\left(x\right)=\frac{d\mu }{d\lambda }\left(x\right)$ denotes its probability density function. The total variational distance [30] of *v* and μ in P(X) is given by (to avoid any confusion, if *S* is a set then $\left|S\right|$ denotes its cardinality).

(1) $$V\left(\mu ,v\right)=\underset{A\in B(X)}{\sup}\left|\mu (A)-v(A)\right|.$$

For μ and *v* belonging to AC(X), if we define the Scheffé set for the pair (μ,v) by

(2) $$A_{\mu ,v}\equiv \left\{x\in X:g_{\mu }\left(x\right)>g_{v}\left(x\right)\right\}\in B\left(X\right),$$

then $V\left(\mu ,v\right)=\mu \left(A_{\mu ,v}\right)-v\left(A_{\mu ,v}\right)$ [30,33].

### 2.2. Fixed-Rate Universal Lossy Source Coding with Memory or Training Data

Let $\left\{X_{n}:n\geq 1\right\}$ be an i.i.d. stochastic process (or stationary and memoryless source), where $X_{i}$ takes values in $X\subset R^{d}$ and has a distribution μ in $F=\left\{\mu_{\theta }:\theta \in \Theta \right\}\subset \mathrm{AC}\left(X\right)$. Θ is in general an index set for F. The problem of lossy source-coding of a finite block of the process $X^{n}=\left(X_{1},...,X_{n}\right)$ reduces to find a mapping (or code) $C^{n}\left(\cdot \right)$ from $X^{n}$ to $S_{n}$, where $S_{n}$ is a finite set. Given a cardinality constraint on $S_{n}$, the design objetive is to make $C^{n}\left(X^{n}\right)$ as close as possible to $X^{n}$ (in average) using for that a distortion function. The standard coding problem assumes the knowledge of μ for finding the optimal code (for any finite block *n*) [1,2,13], as well as for characterizing the fundamental performance limits of this task as *n* goes to infinity [2,24,28,34,35,36].

A more realistic scenario is the universal source coding (USC) problem [2], where the source distribution μ∈F is unknown and a coding scheme needs to be designed optimally for the family F. Here we focus on a specific learning variation of this task introduced by Raginsky in [18], where in addition to the data that needs to be compressed and recovered (with respect to a fidelity criterion), we have a finite number of i.i.d samples following the same distribution μ and that can be used to estimate μ in the encoding process (more details of this approach in Section 2.3). This additional data can be interpreted as memory, training data, or side information about μ available at the encoder because it is data that is not required to be compressed and recovered. The existence of this memory departs from the standard zero-memory setting considered in universal source coding [1]. However, this information can be seen as a realistic assumption in the context of a sequential block by block coding of an infinite sequence, where the data is partitioned into blocks of the same finite length and compressed sequentially block by block. Then in a given stage of this sequential process, the data from previous blocks are available at the encoder (lossless) for the process compressing the current block [18].

More specifically following the fixed-rate block coding and modeling setting introduced by Raginsky in [18], we consider an *n*-block coding scheme with finite memory *m*, where there is a distinction between the data $Z^{m}=\left(Z_{1},\ldots ,Z_{m}\right)$ that is available (as side information) to estimate the source distribution (training data) and the data $X^{n}$ that needs to be encoded and recovered (source or test data), under the important assumption that both data sets are i.i.d. samples of the same unknown probability μ∈F. A systematic exposition of this coding setting and its connection with the classical setting of zero-memory block coding is presented in [18] (Section II). Formally, let us define an (m,n)-block code by the pair

(3) $$C^{m,n}\equiv \left(f:X^{m}\times X^{n}\to S_{n},\;\phi :S_{n}\to {\hat{X}}^{n}\right).$$

Then given a set of training samples $z^{m}\in X^{m}$ and a finite block of the source $x^{n}\in X^{n}$, $C^{m,n}$ is the composition of: a encoding function $f(z^{m},x^{n})$ that maps $x^{n}$ to an element in a finite set $S_{n}$ conditioned on the training data (or memory) represented by $z^{m}$, and a decoding function ϕ(·) that maps a symbol $s\in S_{n}$ into the reproduction points $\Gamma_{C^{m,n}}\equiv \left\{\phi \left(s\right):s\in S_{n}\right\}$ that we called the codebook of $C^{m,n}$. In this context, $\hat{X}$ denotes the reproduction space. As a short-hand, we denote by ${\hat{x}}^{n}=C^{m,n}\left(x^{n}\right)=\phi \left(f\left(z^{m},x^{n}\right)\right)$ the reconstruction of $x^{n}$ obtained by $C^{m,n}$ and its memory $z^{m}$ (for simplicity, the dependency of ${\hat{x}}^{n}$ or $C^{m,n}\left(x^{n}\right)$ on the memory $z^{m}$ will be implicit in the rest of the exposition.). The rate of $C^{m,n}$ in bits-per-letter is given by $R\left(C^{m,n}\right)\equiv \frac{\log_{2}\left|S_{n}\right|}{n}$. In general, it is not possible to recover $x^{n}$ from ${\hat{x}}^{n}$ given the cardinality constraint on $S_{n}$, and thus a single-letter distortion measure $\rho :X\times \hat{X}\to R^{+}$ is used to quantify the *n*-block discrepancy by [24]

(4) $$\rho^{n}\left(x^{n},{\hat{x}}^{n}\right)\equiv \sum_{i=1}^{n}\rho \left(x_{i},{\hat{x}}_{i}\right).$$

Finally considering $X^{n}∼\mu^{n}$ and $Z^{m}∼\mu^{m}$, the average distortion per-letter of $C^{m,n}$ given $Z^{m}$ is

(5) $$D_{\mu }\left(C^{m,n}|Z^{m}\right)\equiv \frac{1}{n}E_{X^{n}∼\mu^{n}}\left(\rho^{n}\left(X^{n},{\hat{X}}^{n}\right)\right),$$

which is a function of $Z^{m}$ and hence the average distortion per-letter of $C^{m,n}$ is

(6) $$D_{\mu }\left(C^{m,n}\right)\equiv E_{Z^{m}∼\mu^{m}}\left(D_{\mu }\left(C^{m,n}|Z^{m}\right)\right).$$

In universal source coding the performance of a code $D_{\mu }\left(C^{m,n}\right)$ is evaluated over a collection of distributions μ∈F and is compared (point-wise) with the best code that can be obtained assuming that μ is known. For this analysis, we need the following definitions:

**Definition** **1** ([18])**.** *For a finite block length n and distribution μ∈F, the n-order operational distortion-rate function of μ at rate R is*

(7) $$D_{\mu }^{n}\left(R\right)\equiv \underset{m\geq 0}{\inf}\underset{\begin{array}{c} C^{m,n}\\ with\;R(C^{m,n})\leq R \end{array}}{\inf}D_{\mu }\left(C^{m,n}\right).$$

*In this context, the operational distortion-rate function (DRF) [2,28] is given by*

(8) $$D_{\mu }\left(R\right)\equiv \lim_{n\to \infty }D_{\mu }^{n}\left(R\right)=\underset{n\geq 1}{\inf}D_{\mu }^{n}\left(R\right).$$

The celebrated Shannon lossy source-coding theorem [27] provides a single letter theoretical characterization for $D_{\mu }\left(R\right)$ in (8) (also known as the Shannon DRF). A nice exposition of this celebrated result can be found in [2,24,28].

It is worth noting that the operational distortion-rate function in (7) is equivalent to the classical zero-memory *n*-order operational distortion-rate function given by $\inf_{C^{0,n}}\left\{D_{\mu }\left(C^{0,n}\right):\mathrm{such}\;\mathrm{that}\;R\left(C^{0,n}\right)\leq R\right\}$ [18] (Lemma 2.1). Then, allowing a nonzero memory (side information at the encoder) does not help in the minimization of the distortion when μ is known.

For the rest of the exposition, we will concentrate on the simple case studied in [18] where n=m (i.e., the block-length is equal to the memory of the code). To be precise about the meaning of universality in this context, we resort to some standard definitions:

**Definition** **2** ([16])**.** *A coding scheme $\left\{C^{n,n}:n\geq 1\right\}$ is weakly minimax universal for the class F at rate R, if ∀μ∈F*

(9) $$\lim_{n\to \infty }D_{\mu }\left(C^{n,n}\right)=D_{\mu }\left(R\right)$$

*and $\lim\sup_{n\to \infty }R\left(C^{n,n}\right)=\lim\sup_{n\to \infty }\frac{\log_{2}\left|S_{n}\right|}{n}\leq R$. Alternatively, the scheme is said to be strongly minimax universal for the class F at rate R if*

(10) $$\lim_{n\to \infty }\underset{\mu \in F}{\sup}\left[D_{\mu }\left(C^{n,n}\right)-D_{\mu }\left(R\right)\right]=0$$

*and $\lim\sup_{n\to \infty }R\left(C^{n,n}\right)\leq R$.*

Decomposing the distortion redundancy in two terms,

(11) $$D_{\mu }\left(C^{n,n}\right)-D_{\mu }\left(R\right)=\left[D_{\mu }\left(C^{n,n}\right)-D_{\mu }^{n}\left(R\right)\right]+\left[D_{\mu }^{n}\left(R\right)-D_{\mu }\left(R\right)\right],$$

the first term $\left[D_{\mu }\left(C^{n,n}\right)-D_{\mu }^{n}\left(R\right)\right]$ is the *n*-order distortion redundancy, which is the discrepancy that can be attributed exclusively to the goodness of the coding scheme. The second term in (11), i.e., $\left[D_{\mu }^{n}\left(R\right)-D_{\mu }\left(R\right)\right]$, has to do with how fast $D_{\mu }^{n}\left(R\right)$ converges to the Shannon DRF as the block length tends to infinity (see further details in [14] (Section III) and references therein). From this observation, we introduce the following definition:

**Definition** **3.** *A coding scheme $\left\{C^{n,n}:n\geq 1\right\}$ is strongly finite-block universal for the class F at rate R if*

(12) $$\lim_{n\to \infty }\underset{\mu \in F}{\sup}\left[D_{\mu }\left(C^{n,n}\right)-D_{\mu }^{n}\left(R\right)\right]=0$$

*and $\lim\sup_{n\to \infty }R\left(C^{n,n}\right)\leq R$.*

Note that if $\left\{C^{n,n}:n\geq 1\right\}$ is strongly minimax universal then it is strongly finite-block universal, but the converse result is not true in general. The missing condition to make these two criteria equivalent is the uniform convergence of $D_{\mu }^{n}\left(R\right)$ to $D_{\mu }\left(R\right)$ in the class F. More discussion about this point in Section 6.

### 2.3. Raginsky’s Two-Stage Joint Universal Coding and Modeling

Motivated by the work of Rissanen [6], Raginsky [18] proposed a two-stage block code with finite memory (training data), with the objective of doing both fixed-rate lossy source coding, and identification of the source distribution at the receiver. More precisely, given $Z^{n}∼\mu_{\theta }^{n}$ and $X^{n}∼\mu_{\theta }^{n}$ (the training and the source-data samples, respectively), an (n,n)-joint coding and modeling rule is given by

(13) $$\begin{array}{cc} C^{n,n}\equiv & \left(f_{n}:X^{n}\to {\tilde{S}}_{n},\phi_{n}:{\tilde{S}}_{n}\to \Theta ,\right.\\ & \left\{\left.f_{n,\tilde{s}}:X^{n}\to S_{n},\phi_{n,\tilde{s}}:S_{n}\to {\hat{X}}^{n};\tilde{s}\in {\tilde{S}}_{n}\right\}\right), \end{array}$$

where $S_{n}$ and ${\tilde{S}}_{n}$ are finite-set functions of *n*. $C^{n,n}$ processes $\left(Z^{n},X^{n}\right)$ in two stages. In the first stage, the pair $\left(f_{n},\phi_{n}\right)$ in (13) uses $Z^{n}$ to do density estimation and finite-rate encoding (quantization) by $f_{n}\left(Z^{n}\right)$, and $\phi_{n}\left(\cdot \right)$ decodes an estimated density in $\left\{\phi_{n}\left(s\right):s\in {\tilde{S}}_{n}\right\}\subset \Theta$. At the end, the first stage provides a quantized estimation of $\mu_{\theta }\in F$ given by

(14) $$\begin{array}{c} {\hat{\theta }}_{n}\left(Z^{n}\right)\equiv \phi_{n}\left(f_{n}\left(Z^{n}\right)\right)\in \Theta . \end{array}$$

Using the index $\tilde{s}=f_{n}\left(Z^{n}\right)\in {\tilde{S}}_{n}$, the second stage of $C^{n,n}$, represented by $\left\{\left(f_{n,s},\phi_{n,s}\right);s\in {\tilde{S}}_{n}\right\}$ in (13), encodes and decodes the source data $X^{n}$ by

(15) $$\begin{array}{c} C^{n,n}\left(X^{n}\right)\equiv \phi_{n,\tilde{s}}\left(f_{n,\tilde{s}}\left(X^{n}\right)\right). \end{array}$$

**Figure 1:**
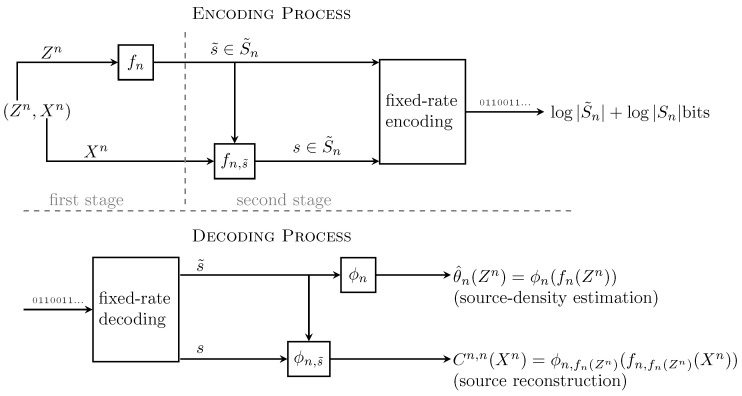
Illustration of Raginsky’s two-stage joint source coding and modeling scheme. Top figure illustrates the coding process and the bottom figure shows the respective decoding process.

In summary, the outcome of the whole encoding process is the concatenation of the bits that represent $f_{n}\left(Z^{n}\right)$ (first-stage bits), and the bits that represent $f_{n,f_{n}\left(Z^{n}\right)}\left(X^{n}\right)$ (second-stage bits). The decoding process, on the other hand, reads the first-stage bits to recover ${\hat{\theta }}_{n}\left(Z^{n}\right)$ and then reads the second-stage bits to recover $C^{n,n}\left(X_{1}^{n}\right)$. (see Figure 1 in which this process is illustrated). The rate (in bits per letter) of $C^{n,n}$ is

(16) $$R\left(C^{n,n}\right)=\frac{\log_{2}\left|{\tilde{S}}_{n}\right|}{n}+\frac{\log_{2}\left|S_{n}\right|}{n}.$$

Based on this two-stage scheme, we could simultaneously achieve source coding and density estimation (modeling) at the decoder. This new joint coding and modeling objective motivates the introduction of the following definition:

**Definition** **4.** *A joint coding and modeling scheme $\left\{C^{n,n}:n\geq 1\right\}$ in (13) is strongly minimax universal for a class of distribution $F=\left\{\mu_{\theta }:\theta \in \Theta \right\}\subset \mathrm{AC}\left(X\right)$ at the rate R>0, if*

- *$\lim_{n\to \infty }\sup_{\mu \in F}\left[D_{\mu }\left(C^{n,n}\right)-D_{\mu }^{n}\left(R\right)\right]=0$,*
- *$\lim_{n\to \infty }\sup_{\mu \in F}E_{Z^{n}∼\mu^{n}}\left(V\left(\mu_{{\hat{\theta }}_{n}\left(Z^{n}\right)},\mu \right)\right)=0$, and*
- *${lim sup}_{n\to \infty }R\left(C^{n,n}\right)\leq R$.*

Consequently, if $\left\{C^{n,n}:n\geq 1\right\}$ is strongly minimax universal for F, it follows that as *n* tends to infinity, density estimation is achieved at the decoder (in expected total variations) and, from the source coding perspective, $\left\{C^{n,n}:n\geq 1\right\}$ is strongly finite-block universal for F in the sense of Definition 3. For the rest of the paper, the strongly minimax universality of Definition 4 will be the main coding and modeling objective.

## 3. Connections with Zero-Rate Density Estimation

This section formalizes a connection between the objective of joint coding and modeling (declared in Definition 4) and a problem of zero-rate density estimation.

### 3.1. Density Estimation with a Rate Constraint

Let us first introduce the problem of rate constrained density estimation. Let $F=\left\{\mu_{\theta }:\theta \in \Theta \right\}\subset \mathrm{AC}\left(X\right)$ be an indexed collection of densities as introduced in Section 2.2.

**Definition** **5.** *An $\left(n,2^{nR}\right)$ learning rule of length n and rate R for F is a pair of functions (f,ϕ), with $f:X^{n}\to S$ and ϕ:S→Θ, where S is a finite set and*

(17) $$\frac{1}{n}\log_{2}\left|\left\{f\left(x^{n}\right):x^{n}\in X^{n}\right\}\right|\;=\frac{1}{n}\log_{2}\left|S\right|=R.$$

*The composition of these two functions $\pi =\phi \circ f:X^{n}\to \Theta$ defines the rate-constrained learning rule for F taking values in the codebook $\left\{\phi (s):s\in S\right\}\subset \Theta$, where $R\left(\pi \right)=\log_{2}\left(\left|S\right|\right)/n$ denotes its description complexity in bits per training sample.*

**Definition** **6.** *The rate R≥0 is achievable for F, if a learning scheme $\Pi =\left\{(f_{n},\phi_{n}):n\geq 1\right\}$ exists such that*

(18) $$\lim_{n\to \infty }\underset{\mu \in F}{\sup}E_{Z^{n}∼\mu^{n}}\left(V\left(\mu_{\pi_{n}\left(Z^{n}\right)},\mu \right)\right)=0\;\mathrm{and}\;\lim\underset{n\to \infty }{\sup}R\left(\pi_{n}\right)\leq R,$$

*where $Z_{1},Z_{2}\ldots$ in the left hand side (LHS) of (18) corresponds to i.i.d. realizations driven by μ∈F. In this case, we say that* Π *is an R-rate uniformly consistent scheme (or estimator) for the class F.*

### 3.2. Main Results

**Proposition** **1.** *If for a given R>0, $\left\{C^{n,n}:n\geq 1\right\}$ is strongly minimax universal for the class F at the rate R (Definition 4), then its induced finite-description learning scheme obtained from the first stage in (13), i.e., $\Pi =\left\{(f_{n},\phi_{n}):n\geq 1\right\}$, is a zero-rate uniformly consistent estimator for F (Definition 6).*

The proof is presented in Section 8.1.

Interestingly, the existence of a zero-rate uniformly consistent scheme for F is also sufficient to achieve the joint coding and modeling objetive (Definition 4) if some mild conditions are adopted from the work in [18]. This is stated in the following result:

**Theorem** **1.** *Let us assume that*

*(i)*  *$\rho :X\times \hat{X}\to R^{+}$ can be expressed by $\rho \left(x,\hat{x}\right)=d{\left(x,\hat{x}\right)}^{p}$ where d(,) is a bounded metric in $X\cup \hat{X}\times X\cup \hat{X}$ with p>0 and*
*(ii)*  *for all μ∈F, for all n≥1, and for all R>0, there exists a (0,n)-block code, say $C_{\mu }^{*n}$, that achieves the n-order operational DRF $D_{\mu }^{n}\left(R\right)$ in (7).*

*Then the existence of a learning scheme $\Pi =\left\{(f_{n},\phi_{n}):n\geq 1\right\}$ that is zero-rate uniformly consistent for F implies that ∀R>0 there exists a joint coding and modeling scheme $\left\{C^{n,n}:n\geq 1\right\}$ that is strongly minimax universal for F at rate R (Definition 4).*

The proof is presented in Section 8.2.

**Remark** **1.** *The construction proposed for $\left\{C^{n,n}:n\geq 1\right\}$ at any rate R>0 (in Section 8.2) using the zero-rate density estimation scheme $\Pi =\left\{\pi_{n}=\phi_{n}\circ f_{n}:n\geq 1\right\}$ satisfies that:*

(19) $$\begin{array}{cc} \underset{\mu \in F}{\sup}\left[D_{\mu }\left(C^{n,n}\right)-D_{\mu }^{n}\left(R\right)\right] & \leq C\cdot \underset{\mu \in F}{\sup}E_{Z^{n}∼\mu^{n}}\left(V\left(\mu_{\pi_{n}\left(Z^{n}\right)},\mu \right)\right)\;\mathrm{and}\; \end{array}$$

(20) $$\begin{array}{cc} R(C^{n,n})-R & \leq R(\pi_{n}), \end{array}$$

*∀n≥1, where C>0 is a constant. It is worth noting that these two inequalities summarize the result in Theorem 1 and, importantly, these two bounds are independent of R.*

**Remark** **2.** *An important consequence of the bounds in (19) and (20) is the fact that constructing a learning scheme $\Pi =\left\{\pi_{n}:n\geq 1\right\}$ with specific rates of convergence for $\sup_{\mu \in F}E\left(V\left(\mu_{\pi_{n}\left(Z^{n}\right)},\mu \right)\right)$ and $R(\pi_{n})$ (as n goes to infinity) produces a joint coding and modeling scheme that achieves a uniform rate of convergence to zero (over F) of the overhead in distortion by (19) and a uniform rate of convergence to zero of the overhead in rate by (20). This observation will be used in all the achievable results presented in Section 4 and Section 5, where, consequently, the problem reduces to determine* Π *and expressions for $\sup_{\mu \in F}E\left(V\left(\mu_{\pi_{n}\left(Z^{n}\right)},\mu \right)\right)$ and $R(\pi_{n})$.*

## 4. Joint Source Coding and Modeling Achievability Results

From the connection with zero-rate density estimation in Section 3, here we present a set of new results for the joint coding and modeling problem of Section 2.3. In these results, the general conditions (i) and (ii) stated in Theorem 1 are assumed.

### 4.1. Main Result: The Skeleton Density Estimator

Let us first introduce some notions from approximation theory [37].

**Definition** **7.** *Let F⊂AC(X) be a class of densities. We say that F is $L_{1}$-totally bounded if for every ϵ>0, there is a finite set of elements $\left\{\mu_{i}:i=1,\ldots ,N\right\}$ in F such that,*

(21) $$\begin{array}{c} F\subset \overset{N}{\underset{i=1}{⋃}}B_{\epsilon }^{V}\left(\mu_{i}\right), \end{array}$$

*where $B_{\epsilon }^{V}\left(\mu \right)\equiv \left\{v\in \mathrm{AC}(X):V(\mu ,v)<\epsilon \right\}$.*

**Definition** **8.** *For F$L_{1}$-totally bounded, let $N_{\epsilon }$ denote the smallest positive integer that achieves the condition in (21). $N_{\epsilon }$ is called the ϵ-covering number of F and $K\left(\epsilon \right)\equiv \log_{2}\left(N_{\epsilon }\right)$ is called the Kolmogorov’s ϵ-entropy of F [30].*

**Definition** **9.** *An ϵ-covering $G_{\epsilon }$ of F such that $\left|G_{\epsilon }\right|=N_{\epsilon }$ is called an ϵ-skeleton of F [29].*

**Theorem** **2.** *There is a strongly minimax universal joint coding and modeling scheme for F at rate R for any rate R>0 if, and only if, F is $L_{1}$-totally bounded.*

The proof is presented in Section 8.3.

The achievability part of the proof of Theorem 2 relies on the adoption of the skeleton estimator [29] (with its minimum distance learning principle in (42)), which is a zero-rate uniformly consistent density estimator for F (Definition 6). Furthermore, Theorem 2 can be complemented saying that the proposed construction $\left\{C^{n,n}:n\geq 1\right\}$ derived from the skeleton estimator satisfies that ($P_{\mu }$ is a short-hand for the process distribution of ${\left(Z_{n}\right)}_{n\geq 1}$ characterized by μ∈F under the i.i.d. assumption.)

(22) $$\begin{array}{c} \lim_{n\to \infty }D_{\mu }\left(C^{n,n}|Z^{n}\right)=D_{\mu }\left(R\right),\;P_{\mu }-almost\;surely, \end{array}$$

(23) $$\begin{array}{c} \lim_{n\to \infty }V\left(\mu_{\pi_{n}\left(Z^{n}\right)},\mu \right)=0,\;P_{\mu }-almost\;surely, \end{array}$$

∀μ∈F. The argument is presented in Appendix A.

### 4.2. Examples of $L_{1}$-Totally Bounded Clases

Knowing specific expressions for $K\left(\epsilon \right)=\log_{2}N_{\epsilon }<\infty$, the skeleton estimator can be optimized selecting its design parameter appropriately. In particular, the sequence ${\left(\epsilon_{n}\right)}_{n\geq 1}$ (see details in Section 8.3) is selected as the solution of the optimal balance between estimation and approximation errors (see (45) in Section 8.3), which is given by $\epsilon_{n}^{*}\equiv \inf\left\{\epsilon >0:\log\left(2N_{\epsilon }^{2}\right)\leq \sqrt{n}\right\}$ [30] (Chapter 7.2). The details of this analysis are presented in Section 8.3 and [30] (Chapter 7). By doing so, an optimized zero-rate skeleton scheme $\Pi =\left\{(f_{\epsilon_{n}^{*}},\phi_{\epsilon_{n}^{*}}),n\geq 1\right\}$, with concrete rate of convergence for $\sup_{\mu \in F}E_{Z^{n}∼\mu^{n}}\left(V\left(\mu_{\pi_{\epsilon_{n}^{*}}\left(Z^{n}\right)},\mu \right)\right)$ and $R(\pi_{\epsilon_{n}^{*}})$, can be obtained. From Remarks 1 and 2, these results imply specific performance results for the induced joint coding and modeling scheme. To illustrate, we present three interesting examples below.

#### 4.2.1. Finite Mixture Classes

Let $F=\left\{\mu_{\theta }:\theta \in \Theta \right\}$ with $\Theta =\left\{\theta \in {\left[0,1\right]}^{d}:\sum_{k=1}^{d}\theta_{i}=1\right\}$ be the class of measures which are a convex combination of $\left\{\mu_{1},\ldots ,\mu_{d}\right\}\subset AC\left(X\right)$, i.e., ∀θ∈Θ, ∀A∈B(X), $\mu_{\theta }\left(A\right)=\sum_{k=1}^{d}\theta_{k}\cdot \mu_{k}\left(A\right)$. F is $L_{1}$-totally bounded with K(ϵ) being O(dlog(1/ϵ)) [30] (Chapter 7.4). From (45) the optimal sequence $\left(\epsilon_{n}^{*}\right)$ is $O(\sqrt{d/n})$ [30], which implies the following finite-rate performance bound [30] (Chapter 7.4):

$$\begin{array}{c} \underset{\theta \in \Theta }{\sup}E\left\{V(\mu_{\pi_{\epsilon_{n}^{*}}\left(Z^{n}\right)},\mu_{\theta })\right\}\leq \sqrt{\frac{Cd\log n}{n}}, \end{array}$$

with *C* a universal non-negative constant. The rate in bits per-sample $R\left(\pi_{\epsilon_{n}^{*}}\right)=K\left(\epsilon_{n}^{*}\right)/n$ is O(logn/n).

#### 4.2.2. Monotone Densities in ${\left[0,1\right]}^{d}$

Let F be the collection of densities with support on ${\left[0,1\right]}^{d}$, monotonically decreasing per coordinate and bounded by a constant L>0. This class is known to be $L_{1}$-totally bounded, and furthermore $K\left(\epsilon \right)\leq \frac{CL^{d}}{\epsilon^{d}}$ [30] (Lemma 7.1), with the constant *C* depending only on *d*. From (45), $\left(\epsilon_{n}^{*}\right)$ being $O(L^{d/d+2}/n^{1/d+2})$ is optimal (please see details in [26,30]) with the following performance bound,

$$\begin{array}{c} \underset{\mu \in F}{\sup}E\left\{V(\mu_{\pi_{\epsilon_{n}^{*}}\left(Z^{n}\right)},\mu )\right\}\leq \frac{CL^{d/d+2}}{n^{1/d+2}}. \end{array}$$

In this case, the rate in bits per sample $R\left(\pi_{\epsilon_{n}^{*}}\right)=K\left(\epsilon_{n}^{*}\right)/n$ is $O(1/n^{2/d+2})$.

#### 4.2.3. *r*-Moment Smooth Class in [0,1]

Let F be the class of densities defined on the bounded support [0,1], with *r* absolutely continuous derivatives (with *r* an integer greater than zero) and satisfying that: ∀f∈F$\int_{\left[0,1\right]}\left|f^{\left(r+1\right)}\right|dx\leq C$ for a constant C>0. This class is $L_{1}$-totally bounded with K(ϵ) being $O(1/\epsilon^{r+1})$ [30] (Chapter 7.6). From (45), the optimal sequence $\left(\epsilon_{n}^{*}\right)$ is $O(1/n^{1/3+r})$, where $\sup_{\mu \in F}E\left\{V(\mu_{\pi_{\epsilon_{n}^{*}}\left(Z^{n}\right)},\mu )\right\}$ is $O(1/n^{1/3+r})$ and the rate in bits per sample $R\left(\pi_{\epsilon_{n}^{*}}\right)=K\left(\epsilon_{n}^{*}\right)/n$ is $O(1/n^{2/3+r})$.

Notably, the last two examples are fully non-parametric, where K(ϵ) is a polynomial function of 1/ϵ. Richer non-parametric examples of $L_{1}$-totally bounded clases of densities, where K(ϵ) is even exponentially in 1/ϵ, are presented in [30] (Chapters 7.6 and 7.8) and its references.

### 4.3. Yatracos Classes with Finite VC Dimension

Looking at the distortion redundancy bound in (19), when F is totally bounded the fastest rate of convergence that could be achieved with the skeleton estimator proposed in Theorem 2 is $O(\sqrt{1/n})$ (see Section 8.3 and the estimation error bound in (45)). In this section, more specific density collections are studied to achieve this best rate $O(\sqrt{1/n})$ for density estimation and distortion redundancy from (19). We follow the path proposed by Yatracos in [38], who explored families of distributions with a finite Vapnik and Chervonenkis (VC) dimension the so-called VC classes [39,40]. Let us first introduce some definitions:

**Definition** **10** ([38])**.** *Let $F=\left\{\mu_{\theta }:\theta \in \Theta \right\}\subset \mathrm{AC}\left(X\right)$ be an indexed collection of densities. The Yatracos class for such a collection is given by*

(24) $$\begin{array}{c} A_{\Theta }=\left\{A_{\theta ,\bar{\theta }}:\theta ,\bar{\theta }\in \Theta ,\theta \neq \bar{\theta }\right\}, \end{array}$$

*where $A_{\theta ,\bar{\theta }}\equiv \left\{x\in X:g_{\mu_{\theta }}\left(x\right)>g_{\mu_{\bar{\theta }}}\left(x\right)\right\}\in B\left(X\right)$ is the Scheffé set of $\mu_{\theta }$ with respect to $\mu_{\bar{\theta }}$, as defined in (2).*

**Theorem** **3.** *Let us assume that*

*(i)*  F is $L_{1}$-totally bounded,
*(ii)*  the Yatracos class $A_{\Theta }$ has a finite VC dimension (Definition A1 in Appendix B), and
*(iii)*  the Kolmogorov’s entropy of F associated with the sequence $\epsilon_{n}=1/\sqrt{n}$ grows strictly sub-linearly, i.e., $\log_{2}\left(N_{1/\sqrt{n}}\right)$ is o(n),

*then there is a zero-rate density estimator scheme $\Pi =\left\{(f_{n},\phi_{n}):n\geq 1\right\}$ for F such that*

$$\begin{array}{c} \underset{\mu \in F}{\sup}E_{Z^{n}∼\mu^{n}}\left\{V(\mu_{\pi_{n}\left(Z^{n}\right)},\mu )\right\}\;\mathrm{is}\;O\left(1/\sqrt{n}\right), \end{array}$$

*where $\pi_{n}\left(Z^{n}\right)=\phi_{n}\left(f_{n}\left(Z^{n}\right)\right)$ is the skeleton estimator in (42) with $\epsilon_{n}=1/\sqrt{n}$. Furthermore,* Π *is also a zero-rate strongly consistent density estimator where ∀μ∈F*

$$V\left(\mu_{\pi_{n}\left(Z^{n}\right)},\mu \right)\;\mathrm{is}\;O\left(\sqrt{\log n/n}\right),\;P_{\mu }-almost\;surely.$$

The proof is presented in Section 8.4.

From Definition 7, $\log_{2}\left(N_{\epsilon }\right)$ is inversely proportional to ϵ. In fact, depending of how rich F is, $\log_{2}\left(N_{\epsilon }\right)$ can go from being O(log1/ϵ), passing from being polynomial in 1/ϵ, to being $O(e^{1/\epsilon })$ (see a number of examples in [30] (Chapter 7) and its references). Then the role of (iii) in the statement of Theorem 3 is to bound how fast $N_{\epsilon }$ should tend to infinity as ϵ goes to zero, to guarantee a zero-rate in the skeleton learning scheme. It is simple to show that $N_{\epsilon }$ being $O(e^{{\left(1/\epsilon \right)}^{q}})$ with q∈[0,2) is sufficient to achieve that $\log_{2}\left(N_{1/\sqrt{n}}\right)$ is o(n). This is a condition satisfied by a rich collection of $L_{1}$-totally bounded classes in AC(X). Concrete examples are presented in [30] (Chapter 7).

## 5. The Parametric Scenario

The results presented so far are of theoretical interest because they rely on the skeleton estimator that is constructed from the skeleton covering of F (see Definition 9), which is unknown in practice. Moving towards making the zero-rate skeleton learning scheme of practical interest, we revisit the important parametric scenario in which Θ, the index set of F, is a compact set contained in a finite-dimensional Euclidean space $R^{k}$. Interestingly, in this context we can consider a practical covering of F induced by the uniform partition of the parameter space Θ, as used in [18]. Unlike [18], where a minimum-distance estimate is first found and then quantized, here we first quantize the space Θ and then find the minimum-distance estimate among a finite collection of candidates (i.e., over a finte number of prototypes in Θ). Some assumptions will be needed.

**Definition** **11** ([18])**.** *Let $F=\left\{\mu_{\theta }:\theta \in \Theta \right\}$ with $\Theta \subset R^{k}$. Let $I_{F}:\Theta \to F$ be the index function of F that maps θ to $\mu_{\theta }$. $I_{F}$ is said to be locally uniformly Lipschitz, if there exists r>0 and m>0, such ∀θ∈Θ, $\forall \phi \in B_{r}\left(\theta \right)$,*

(25) $$V\left(\mu_{\theta },\mu_{\phi }\right)\leq m\left|\left|\theta -\phi \right|\right|,$$

*where $B_{r}\left(\theta \right)\subset \Theta$ denotes the ball of radius r (with respect to the Euclidean norm in $R^{k}$) centered at θ.*

The following lemma shows that F is $L_{1}$-totally bounded under some parametric assumptions.

**Lemma** **1.** *Let $F=\left\{\mu_{\theta }:\theta \in \Theta \right\}\subset P\left(X\right)$ with $\Theta \subset R^{k}$. If* Θ *is bounded (∃L>0 such that $\Theta \subset {⨂}_{i=1}^{k}\left[-L,L\right]$) and the mapping $I_{F}:\Theta \to F$ is locally uniformly Lipschitz (Definition 11), then F is $L_{1}$-totally bounded. Furthermore, $N_{\epsilon }$ is $O(1/\epsilon^{k})$ for this family.*

The proof is presented in Section 8.5.

It is important to note that the ϵ-covering of F used in the proof of Lemma 1 to derive an upper bound for $N_{\epsilon }$ is practical (see Appendix C). This offers the possibility of implementing a practical skeleton estimator, which is the focus of the following result.

### The Practical Skeleton Estimator

Under the assumptions of Lemma 1, let $\left({\tilde{f}}_{n,\epsilon },{\tilde{\phi }}_{n,\epsilon }\right)$ denote the learning rule of length *n* associated with the minimum-distance principle in (42) with parameter ϵ (see details in Section 8.3), where instead of using the ϵ-skeleton $G_{\epsilon }$ of F (in Definition 9), the implementable (see Appendix C) ϵ-covering of Θ presented in the proof of Lemma 1 is used. This practical ϵ-covering is denoted by ${\tilde{G}}_{\epsilon }$ (by definition, $N_{\epsilon }=\left|G_{\epsilon }\right|\leq \left|{\tilde{G}}_{\epsilon }\right|={\tilde{N}}_{\epsilon }∼O\left(1/\epsilon^{k}\right)$, this last part from Lemma 1.). With this, let $\tilde{\Pi }\left({\left(\epsilon_{n}\right)}_{n\geq 1}\right)\equiv \left\{({\tilde{f}}_{n,\epsilon_{n}},{\tilde{\phi }}_{n,\epsilon_{n}}):n\geq 1\right\}$ denote our practical learning scheme indexed by the precision numbers ${\left(\epsilon_{n}\right)}_{n\geq 1}\in {\left(R^{+}\right)}^{N}$. We are in a position to integrate Theorem 3 and Lemma 1 to state the following:

**Theorem** **4.** *Under the assumptions of Lemma 1, the practical skeleton estimator $\tilde{\Pi }\left({\left(\epsilon_{n}\right)}_{n\geq 1}\right)$ with $\epsilon_{n}^{*}=1/\sqrt{n}$ satisfies that*

(26) $$\begin{array}{c} \underset{\mu_{\theta }\in F}{\sup}E_{Z^{n}∼\mu^{n}}\left\{V(\mu_{{\tilde{\pi }}_{n,\epsilon_{n}^{*}}\left(Z^{n}\right)},\mu_{\theta })\right\}\;\mathrm{is}\;O\left(\sqrt{\log n/n}\right),\;\mathrm{and}\;R\left({\tilde{\pi }}_{n,\epsilon_{n}^{*}}\right)\;\mathrm{is}\;O\left(\log n/n\right), \end{array}$$

*where ${\tilde{\pi }}_{n,\epsilon }\left(Z^{n}\right)\equiv {\tilde{\phi }}_{n,\epsilon }\left({\tilde{f}}_{n,\epsilon }\left(Z^{n}\right)\right)$.*

*In addition, if the Yatracos collection $A_{\Theta }=\left\{A_{\theta ,\bar{\theta }}:\theta ,\bar{\theta }\in \Theta ,\theta \neq \bar{\theta }\right\}$ has a finite VC dimension equal to J, then*

(27) $$\begin{array}{c} \underset{\mu_{\theta }\in F}{\sup}E_{Z^{n}∼\mu^{n}}\left\{V(\mu_{{\tilde{\pi }}_{n,\epsilon_{n}^{*}}\left(Z^{n}\right)},\mu_{\theta })\right\}\;\mathrm{is}\;O\left(1/\sqrt{n}\right),\;\mathrm{and}\;R\left({\tilde{\pi }}_{n,\epsilon_{n}^{*}}\right)\;\mathrm{is}\;O\left(\log n/n\right). \end{array}$$

The proof is presented in Section 8.6.

When $X\subset R^{d}$, Raginsky [18] showed that the finite VC dimension assumption of Theorem 4 is satisfied by the class of mixture families presented in Section 4.2.1 and a rich collection of exponential families of the form $F=\left\{\mu_{\theta }:\theta \in \Theta \right\}\subset P\left(X\right)$ with $\frac{d\mu_{\theta }}{d\lambda }\left(x\right)=f\left(x\right)\cdot e^{\sum_{i=1}^{k}\theta_{i}h_{i}\left(x\right)-g\left(\theta \right)},\;\forall x\in X$, where f(x) is a reference density, $\left\{h_{i}\left(\cdot \right):i=1,\ldots ,k\right\}$ is a set of arbitrary real-valued functions, g(θ) is a normalization constant ($g\left(\theta \right)=\ln\int_{X}e^{\sum_{i=1}^{k}\theta_{i}h_{i}\left(x\right)}f\left(x\right)dx$ see details in [18] (Section V)), and Θ is a compact subset of $R^{k}$ (see details in [18] (Section V)).

## 6. Summary of the Results

We summarize the results of the proposed zero-rate density estimation approach adopted for the problem of joint fixed-rate lossy source coding and modeling of continuous memoryless sources.

- Proposition 1 and Theorem 1 formalize the interplay between the two-stage joint fixed-rate coding and modeling objective and the problem of zero-rate uniformly consistent (in expected total variation) density estimation.
- Theorem 2 establishes a necessary and sufficient condition on a family of densities for the existence of a strongly minimax joint coding and modeling scheme achieving both source coding and model identification objectives (Definition 4). The result is obtained for the rich non-parametric collection of $L_{1}$-totally bounded densities.
- For the modeling stage, we propose using the skeleton estimator, which first quantizes the data and then finds the minimum-distance decision on this finite set of density candidates (42). This is a practical solution in the sense that the inference (minimization) is carried out over a finite set.
- By introducing combinatorial regularity conditions on the family of distributions $F=\left\{\mu_{\theta }:\theta \in \Theta \right\}$, the skeleton scheme achieves $O(1/\sqrt{n})$ rate of convergence in the *n*-order distortion redundancy, and the same rate in the expected total variational distance for the modeling part (Theorem 3).
- Finally, for a relevant parametric setting, a practical skeleton-based joint coding and modeling scheme is proposed that achieves a rate of $O(1/\sqrt{n})$ for the *n*-order distortion redundancy (Theorem 4). This rate is slightly better than the $O(\sqrt{\log n/n})$ achieved in [18] under the same rate overhead of O(log(n)/n). Furthermore, Theorem 4 removes the finite-VC-dimension assumption over the Yatracos class $A_{\Theta }$ considered in [18] (Theorem 3.2), while achieving the same performance rates in terms of *n*-order distortion redundancy $O(\sqrt{\log n/n})$, uniform expected risk to learn the density $O(\sqrt{\log n/n})$, and rate overhead O(logn/n).

Concerning the last parametric result, we note that the result in [18] can be improved by the adoption of Dudley’s entropy bound [41], which would yield the same asymptotic rate reported in this work for the *n*-order distortion redundancy.

A final remark is that under the bounded distortion metric assumption of Theorem 1 condition (i), Linder et al. [14] (Theorem 2) showed that ∀θ∈Θ, and for every R>0 such that $D_{\mu_{\theta }}\left(R\right)>0$, there is a constant $K_{\theta }\left(R\right)>0$ such that

(28) $$\begin{array}{c} D_{\mu_{\theta }}^{n}\left(R\right)-D_{\mu_{\theta }}\left(R\right)\leq \left(K_{\theta }\left(R\right)+r_{n}\right)\sqrt{\frac{\log n}{n}}, \end{array}$$

where $\left(r_{n}\right)$ is a sequence that converges to zero (o(1)) uniformly in Θ. This result offers a rate of convergence of the *n*-order operational distortion-rate function to the Shannon DRF as the block length tends to infinity. In view of (11), we can adopt this result in Theorems 3 and 4, to say that the average distortion of the respective joint coding and modeling schemes at rate *R*, i.e., $D_{\mu }\left(C^{n,n}\right)$, convergences to the Shannon DRF $D_{\mu }\left(R\right)$ as $O(\sqrt{\frac{\log n}{n}})$ point-wise ∀μ∈F. Therefore in the process of comparing $D_{\mu }\left(C^{n,n}\right)$ with the Shannon DR function, we lose the $O(\sqrt{1/n})$ rate of convergence.

## 7. Conclusions

This work revisits the problem of fixed-rate universal lossy source coding and model identification with training data proposed in [18] from a learning perspective. Remarkably, we found that the problem is equivalent to the problem of density estimation of the source distribution with some concrete but non-conventional operational data-rate constraints in bits per sample. This learning problem can be seen as the task of estimating and encoding the distribution of samples with a zero-rate in bits per sample, while achieving a consistent estimation in expected total variations of the distribution after the decoding process. From our perspective, the rate-constraint density estimation problem is interesting in itself and can have relevant applications in other contexts such as distributed learning scenarios and sensor network problems.

Importantly for the joint coding and modeling problem, the connection with density estimation provides a context for the use of the skeleton estimator proposed by Yatracos in [29]. We highlight two important implications from its use. First, we extend results about minimax universality from the parametric context explored in [30] to the rich non-parametric family of $L_{1}$-totally bounded densities [26,30]. This result significantly expands the contexts where the joint model and coding objective can be achieved. We illustrated this with some examples in Section 4.2 and many more can be found in the literature of density estimation [26,30].

Second, in the parametric case studied in [18], we were able to remove some of the assumptions and obtain not only the same performance result in terms of rate of convergence of the *n*-order distortion redundancy but also slightly better convergence results. Therefore, the Skeleton estimator, though essentially a non-parametric learning scheme, is shown to be instrumental in enriching the applicability of the joint coding and modeling framework.

## 8. Proofs of Results

### 8.1. Proposition 1

**Proof.**  The fact that Π is uniformly consistent for F is directly from Definition 4. On the other hand, the rate of $\pi_{n}=\phi_{n}\circ f_{n}$ is $R\left(\pi_{n}\right)=\frac{1}{n}\log_{2}\left|{\tilde{S}}_{n}\right|$. From the definition of $D_{\mu }^{n}\left(R\right)$, it is simple to show from the strict monotonicity of $D_{\mu }\left(R\right)$ that in order for $\lim_{n\to \infty }\sup_{\mu \in F}\left[D_{\mu }\left(C^{n,n}\right)-D_{\mu }^{n}\left(R\right)\right]=0$, it is required that ${lim sup}_{n\to \infty }\frac{1}{n}\log\left|S_{n}\right|>R-\epsilon$ for any ϵ>0. Then, from (16), and since $\log|{\tilde{S}}_{n}|/n=R\left(\pi_{n}\right)$, ${lim sup}_{n\to \infty }R\left(C^{n,n}\right)\leq R$ implies that $\lim_{n\to \infty }R\left(\pi_{n}\right)=0$. ☐

### 8.2. Theorem 1

**Proof.**  The proof builds upon the ideas elaborated in [18] (Theorem 3.2, p. 3065). Let us consider an arbitrary R>0 and let $\Pi =\left\{(f_{n},\phi_{n}):n\geq 1\right\}$ be the zero-rate learning scheme of the assumption. Using Π, let us construct the joint coding and modeling rule of length *n* by:

(29) $$\begin{array}{cc} C^{n,n}= & \left(f_{n}:X^{n}\to {\tilde{S}}_{n},\phi_{n}:{\tilde{S}}_{n}\to \Theta ,\right.\\ & \left\{\left.f_{n,\tilde{s}}:X^{n}\to S_{n},\phi_{n,\tilde{s}}:S_{n}\to {\hat{X}}^{n}:\tilde{s}\in {\tilde{S}}_{n}\right\}\right). \end{array}$$

Concerning the first stage of $\left\{C^{n,n}:n\geq 1\right\}$, it is induced directly from the coding-decoding rules of Π. For the second stage, ∀n≥1, $\forall \tilde{s}\in {\tilde{S}}_{n}$ the pair $\left(f_{n,\tilde{s}},\phi_{n,\tilde{s}}\right)$ is picked such that $C_{\mu_{\theta_{n,\tilde{s}}}}^{*n}=\phi_{n,\tilde{s}}\circ f_{n,\tilde{s}}$, which is the optimal *n*-block code that achieves $D_{\mu_{\theta_{n,\tilde{s}}}}^{n}\left(R\right)$ (from the hypothesis in (ii)), with $\theta_{n,\tilde{s}}\equiv \phi_{n}\left(f_{n}\left(\tilde{s}\right)\right)$ short-hand for the reproduction codeword induced from the first stage-pair $\left(f_{n},\phi_{n}\right)$, and $S_{n}$ satisfying the *R*-rate constraint, i.e., $\left|S_{n}\right|=2^{nR}$. From construction and the fact that Π has zero-rate,

$$\lim_{n\to \infty }R\left(C^{n,n}\right)=R+\lim_{n\to \infty }\log_{2}\left|{\tilde{S}}_{n}\right|/n=R,$$

then $\left\{C^{n,n}:n\geq 1\right\}$ satisfies the rate condition. On the other hand, based on the assumption that Π is zero-rate uniformly consistent, it follows that

(30) $$\lim_{n\to \infty }\underset{\mu \in F}{\sup}E\left(V\left(\mu_{{\hat{\theta }}_{n}\left(Z^{n}\right)},\mu \right)\right)=0,$$

where ${\hat{\theta }}_{n}\left(Z^{n}\right)=\phi_{n}\left(f_{n}\left(Z^{n}\right)\right)$. Then $\left\{C^{n,n}:n\geq 1\right\}$ achieves the modeling objective. Concerning the coding objective, we use the following key result:

**Lemma** **2** ([18] (Lemma C.1))**.** *Let P and Q be two probability measures in (X,B(X)). Let $C^{n}=\left(f,\phi \right)$ be a zero-memory n-block coder with the nearest neighbor property (i.e., $C^{n}$ is nearest neighbor if, $\forall x_{1}^{n}\in X^{n}$, $\phi \left(f\left(x_{1}^{n}\right)\right)=\arg\min_{{\hat{x}}_{1}^{n}\in \Gamma_{C^{n}}}\rho \left(x_{1}^{n},{\hat{x}}_{1}^{n}\right)$ with $\Gamma_{C^{n}}$ the reproduction codebook of $C^{n}$.). If we denote the performance of $C^{n}$ ($C^{n}=\phi \circ f$) with respect to P by*

(31) $$D_{P}\left(C^{n}\right)\equiv \frac{1}{n}E_{X^{n}∼P^{n}}\left(\rho (C^{n}\left(X^{n}\right),X^{n})\right),$$

*where $P^{n}$ denotes the product measure with marginal P in $\left(X^{n},B\left(X^{n}\right)\right)$, and ρ satisfies the condition i) of Theorem 1 and is bounded by $d_{max}$, then*

(32) $$\left|D_{P}{\left(C^{n}\right)}^{1/p}-D_{Q}{\left(C^{n}\right)}^{1/p}\right|\leq 2^{1/p}d_{max}\cdot V\left(P,Q\right).$$

*Furthermore, the inequality can be extended for the n-order operational distortions in (7), i.e.,*

(33) $$\left|D_{P}^{n}{\left(R\right)}^{1/p}-D_{Q}^{n}{\left(R\right)}^{1/p}\right|\leq 2^{1/p}d_{max}\cdot V\left(P,Q\right),$$

*∀R>0.*

Let us work with the following distortion redundancy,

$$\begin{array}{cc} D_{\mu }\left(C^{n,n}|Z^{n}\right)-D_{\mu }^{n}\left(R\right)= & \left[\frac{1}{n}E_{X^{n}∼P_{\mu }^{n}}\left(\rho^{n}\left(X^{n},C^{n,n}\left(X^{n}\right)\right)\right)-D_{\mu_{{\hat{\theta }}_{n}\left(Z^{n}\right)}}^{n}\left(R\right)\right]+ \end{array}$$

(34) $$\begin{array}{c} \left[D_{\mu_{{\hat{\theta }}_{n}\left(Z^{n}\right)}}^{n}\left(R\right)-D_{\mu }^{n}\left(R\right)\right] \end{array}$$

(35) $$\begin{array}{c} \;\leq D_{\mu }\left(C_{\mu_{{\hat{\theta }}_{n}\left(Z^{n}\right)}}^{*n}\right)-D_{\mu_{{\hat{\theta }}_{n}\left(Z^{n}\right)}}^{n}\left(R\right)+2^{1/p}d_{max}\cdot V\left(\mu_{{\hat{\theta }}_{n}\left(Z^{n}\right)},\mu \right) \end{array}$$

$$\begin{array}{c} =D_{\mu }\left(C_{\mu_{{\hat{\theta }}_{n}\left(Z^{n}\right)}}^{*n}\right)-D_{\mu_{{\hat{\theta }}_{n}\left(Z^{n}\right)}}\left(C_{\mu_{{\hat{\theta }}^{n}\left(Z^{n}\right)}}^{*n}\right) \end{array}$$

(36) $$\begin{array}{c} +2^{1/p}d_{max}\cdot V\left(\mu_{{\hat{\theta }}_{n}\left(Z^{n}\right)},\mu \right) \end{array}$$

(37) $$\begin{array}{c} \leq 2^{1/p+1}d_{max}\cdot V\left(\mu_{{\hat{\theta }}_{n}\left(Z^{n}\right)},\mu \right). \end{array}$$

For the first equality we use (5). The inequality in (35) is from the definition in (31) and (33), and the equality in (36) is from the construction of $C_{\mu_{{\hat{\theta }}_{n}\left(Z^{n}\right)}}^{*n}$ which is *n*-operational optimal for the distribution $\mu_{{\hat{\theta }}_{n}\left(Z^{n}\right)}$ at rate *R*. Finally, (37) is from (32).

Concluding, $D_{\mu }\left(C^{n,n}|Z^{n}\right)-D_{\mu }^{n}\left(R\right)$ is random (a measurable function of $Z^{n}$) and dominated by $V(\mu_{{\hat{\theta }}_{n}\left(Z^{n}\right)}\mu )$. Hence taking the expected value (with respect to $Z^{n}$) on both sides of this inequality (see (6)), we have the uniform convergence in (30) implying that

(38) $$\lim_{n\to \infty }\underset{\mu \in F}{\sup}\left[D_{\mu }\left(C^{n,n}\right)-D_{\mu }^{n}\left(R\right)\right]=0,$$

and then the coding objective is achieved. ☐

### 8.3. Theorem 2

**Proof.**  Let us first assume that F is $L_{1}$-totally bounded and prove the direct part of the statement. We adopt the skeleton estimate proposed by Yatracos [29] and extended by Devroye et al. [42,43] (a complete presentation can be found in [30] (Chapter 7)). For any arbitrary ϵ>0, let us consider the ϵ-skeleton $G_{\epsilon }=\left\{\mu_{\theta_{i}^{\epsilon }}:i=1,\ldots ,N_{\epsilon }\right\}$ of F. We use $g_{\theta_{i}^{\epsilon }}\left(x\right)\equiv \frac{d\mu_{\theta_{i}^{\epsilon }}}{d\lambda }\left(x\right)$ as short-hand for the *i*-th pdf in $G_{\epsilon }$, and we define

$$\Theta_{\epsilon }\equiv \left\{\theta_{i}^{\epsilon }:i=1,\ldots ,N_{\epsilon }\right\}\subset \Theta$$

to represent the index set of $G_{\epsilon }$. Let us consider the Yatracos class of $G_{\epsilon }$ given by [30]

(39) $$\begin{array}{c} A_{\epsilon }\equiv \left\{A_{i,j}^{\epsilon },A_{j,i}^{\epsilon }:1\leq i<j\leq N_{\epsilon }\right\}, \end{array}$$

where $A_{i,j}^{\epsilon }=\left\{x\in X:g_{\theta_{i}^{\epsilon }}\left(x\right)>g_{\theta_{j}^{\epsilon }}\left(x\right)\right\}\in B\left(X\right)$ is the Scheffé set of $\mu_{\theta_{i}^{\epsilon }}$ with respect to $\mu_{\theta_{j}^{\epsilon }}$ in (2) [30,33]. Hence, given i.i.d. realizations $X_{1},...,X_{n}$ with $X_{i}∼\mu_{\theta }$ ($\mu_{\theta }\in F$), let us propose the encoder-decoder pair $\left(f_{n,\epsilon },\phi_{n,\epsilon }\right)$ associated with $A_{\epsilon }$ by,

(40) $$\begin{array}{cc} f_{n,\epsilon }\left(X^{n}\right) & \equiv \arg\min_{i\in \left\{1,...,N_{\epsilon }\right\}}\underset{B\in A_{\epsilon }}{\sup}\left|\mu_{\theta_{i}^{\epsilon }}\left(B\right)-{\hat{\mu }}_{n}\left(B\right)\right|\in \left[N_{\epsilon }\right], \end{array}$$

(41) $$\begin{array}{cc} \phi_{n,\epsilon }\left(i\right) & \equiv \theta_{i}^{\epsilon }\in \Theta_{\epsilon }\subset \Theta , \end{array}$$

where ${\hat{\mu }}_{n}\left(B\right)=\sum_{j=1}^{n}1_{B}\left(X_{j}\right)$ is the standard empirical distribution. In this context,

(42) $$\begin{array}{c} {\hat{\theta }}_{\epsilon }\left(X^{n}\right)=\phi_{n,\epsilon }\left(\left(f_{n,\epsilon }\left(X^{n}\right)\right)\right)=\arg\min_{\theta_{i}^{\epsilon }\in \Theta_{\epsilon }}\underset{B\in A_{\epsilon }}{\sup}\left|\mu_{\theta_{i}^{\epsilon }}\left(B\right)-{\hat{\mu }}_{n}\left(B\right)\right|, \end{array}$$

is the well-known skeleton estimate [29]. ${\hat{\theta }}_{\epsilon }\left(X_{1}^{n}\right)$ is the minimum-distance approximation of ${\hat{\mu }}_{n}$ with elements of $G_{\epsilon }$ [29,30], adopting the measure in the right-hand-side of (42) that is reminiscent of the total variational distance in (1). In order to choose a sequence ${\left(\epsilon_{n}\right)}_{n\geq 1}$, we consider the following performance bound.

**Lemma** **3** ([30] (Theorem 6.3))**.** *For any μ∈F,*

(43) $$\begin{array}{c} V\left(\mu_{{\hat{\theta }}_{\epsilon }\left(X^{n}\right)},\mu \right)\leq 3\min_{v\in G_{\epsilon }}V\left(v,\mu \right)+4\underset{B\in A_{\epsilon }}{\sup}\left|{\hat{\mu }}_{n}\left(B\right)-\mu \left(B\right)\right|. \end{array}$$

Equation (43) is valid for any ϵ>0 and, consequently, it provides a trade-off between an approximation error term and an estimation error term. The approximation error is $\min_{v\in G_{\epsilon }}V\left(v,\mu \right)$, which is bounded by the definition of $G_{\epsilon }$. For the estimation error, on the other hand, Yatracos proposed the use of Hoeffding’s inequality [44] to obtain that ∀μ∈P(X) [30] (Theorem 7.1),

(44) $$\begin{array}{c} E_{X^{n}∼\mu^{n}}\left(\underset{B\in A_{\epsilon }}{\sup}\left|{\hat{\mu }}_{n}\left(B\right)-\mu \left(B\right)\right|\right)\leq \sqrt{\frac{\log(2N_{\epsilon }^{2})}{2n}}. \end{array}$$

Using (44) in (43), it follows that, $\sup_{\mu_{\theta }\in F}E\left\{V(\mu_{{\hat{\theta }}_{\epsilon }\left(X^{n}\right)},\mu_{\theta })\right\}\leq 3\epsilon +\sqrt{\frac{8\log(2N_{\epsilon }^{2})}{n}}$. This last expression is distribution-free and it is valid if the approximation fidelity ϵ is a chosen function of *n* [30]. Consequently, for any sequence ${\left(\epsilon_{n}\right)}_{n\geq 1}$,

(45) $$\begin{array}{c} \underset{\mu_{\theta }\in F}{\sup}E\left\{V(\mu_{{\hat{\theta }}_{\epsilon_{n}}\left(X^{n}\right)},\mu_{\theta })\right\}\leq 3\epsilon_{n}+\sqrt{\frac{8\log(2N_{\epsilon_{n}}^{2})}{n}}, \end{array}$$

for all n≥1. Hence, we consider $\epsilon_{n}^{*}\equiv \inf\left\{\epsilon >0:\log\left(2N_{\epsilon }^{2}\right)\leq \sqrt{n}\right\}$ proposed in [30] (Chapter 7.2), which is well-defined and converges to zero as *n* tends to infinity. Consequently from (45), $\lim_{n\to \infty }\sup_{\mu_{\theta }\in F}E\left\{V(\mu_{{\hat{\theta }}_{\epsilon_{n}^{*}}\left(X^{n}\right)},\mu_{\theta })\right\}=0$. Then the learning scheme $\Pi \left({\left(\epsilon_{n}^{*}\right)}_{n\geq 1}\right)\equiv \left\{(f_{n,\epsilon_{n}^{*}},\phi_{n,\epsilon_{n}^{*}}):n\geq 1\right\}$ satisfies the learning requirement in Definition 6, where in particular $R\left(\phi_{n,\epsilon_{n}^{*}}\circ f_{n,\epsilon_{n}^{*}}\right)=\frac{\log_{2}\left(N_{\epsilon_{n}^{*}}\right)}{n}$ is $O(1/\sqrt{n})$ by construction. To conclude the argument of this part (i.e., presenting the construction of the second stage of a joint coding & modeling scheme), we adopt the result and the construction presented in the proof of Theorem 1 (see Remark 1 for details). This result implies that ∀R>0 there is a strongly minimax universal joint coding and modeling scheme for F at rate *R*.

For the other implication (the converse part of the statement), let us fix R>0 and assume that we have a joint coding & modeling scheme that is strongly minimax universal (Definition 4) for F at rate *R*. Then from Proposition 1, we have a learning scheme $\Pi =\left\{(f_{n},\phi_{n}):n\geq 1\right\}$ such that $\lim_{n\to \infty }R\left(\pi_{n}=\phi_{n}\circ f_{n}\right)=0$ and

(46) $$\begin{array}{c} \lim_{n\to \infty }\underset{\mu \in F}{\sup}E_{P_{\mu }^{n}}\left\{V(\mu_{\pi_{n}\left(X^{n}\right)},\mu )\right\}=0. \end{array}$$

For the learning rule of length *n*, we have its reproduction codebook that we denote by $\Theta^{n}\equiv \left\{\theta_{j}^{n}:j=1,\ldots ,2^{nR(\pi_{n})}\right\}\subset \Theta$. Let us define the minimum-distance oracle solution in $\Theta^{n}$ by

(47) $$\begin{array}{c} {\tilde{\theta }}_{n}\left(\mu \right)=\arg\underset{\theta \in \Theta^{n}}{\inf}V\left(\mu_{\theta },\mu \right). \end{array}$$

From (46), we have that $\lim_{n\to \infty }\sup_{\mu \in F}V\left(\mu_{{\tilde{\theta }}_{n}\left(\mu \right)},\mu \right)=0$. In other words, ∀ϵ>0, there exists N(ϵ)<∞, such that for all n≥N(ϵ), $V(\mu_{{\tilde{\theta }}_{n}\left(\mu \right)},\mu )<\epsilon$ uniformly for every element μ∈F. This means that ∀ϵ>0 there exists N(ϵ)<∞, such that for any arbitrary $\bar{n}>N\left(\epsilon \right)$, $F\subset {⋃}_{\theta \in \Theta^{\bar{n}}}B_{\epsilon }\left(\mu_{\theta }\right)$, where by construction $\left|\Theta^{\bar{n}}\right|<\infty$. Then F is totally bounded, which concludes the proof. ☐

### 8.4. Theorem 3

**Proof.**  From Lemma 3, for any arbitrary sequence ${\left(\epsilon_{n}\right)}_{n\geq 1}$

(48) $$V\left(\mu_{{\hat{\theta }}_{\epsilon_{n}}\left(X^{n}\right)},\mu_{\theta }\right)\leq 3\epsilon_{n}+4\underset{B\in A_{\epsilon_{n}}}{\sup}\left|{\hat{\mu }}_{n}\left(B\right)-\mu_{\theta }\left(B\right)\right|.$$

with $A_{\epsilon_{n}}$ the Yatracos class of the skeleton $G_{\epsilon_{n}}$. It is clear that ∀ϵ>0, $A_{\epsilon }\subset A_{\Theta }$. Then by monotonicity $E\left(\sup_{B\in A_{\epsilon }}\left|{\hat{\mu }}_{n}\left(B\right)-\mu \left(B\right)\right|\right)\leq E\left(\sup_{B\in A_{\Theta }}\left|{\hat{\mu }}_{n}\left(B\right)-\mu \left(B\right)\right|\right)$, for all ϵ>0 and for any distribution μ∈P(X). Here is where we use the assumption that $A_{\Theta }$ has finite VC dimension *J*, which implies from [30] (Theorem 3.1) that

(49) $$\underset{\mu \in F}{\sup}E\left(\underset{B\in A_{\Theta }}{\sup}\left|{\hat{\mu }}_{n}\left(B\right)-\mu \left(B\right)\right|\right)\leq c\sqrt{\frac{J}{n}}$$

for some constant c>0. Substituting this result in (48), the argument concludes by replacing $\left(\epsilon_{n}\right)=\left(1/\sqrt{n}\right)$, a solution which achieves the intended rate of convergence for $\sup_{\mu_{\theta }\in F}E\left\{V(\mu_{{\hat{\theta }}_{1/\sqrt{n}}\left(X^{n}\right)},\mu_{\theta })\right\}$. Finally, the rate of the learning rule is $\frac{\left\lceil \log_{2}\left(N_{1/\sqrt{n}}\right)\right\rceil }{n}$, which tends to zero by the last hypothesis.

For the almost-sure convergence part if $\epsilon_{n}^{*}=\frac{1}{\sqrt{n}}$, it is sufficient to show that the second term in the right hand side (RHS) of (48) is $O(\sqrt{\log n/n})$$P_{\mu }$-almost surely. From the fact that $A_{\Theta }$ has finite VC dimension (Definition A1), and from the classical VC inequality [30] (Corollary 4.1 and Theorem 3.1) and [45] (Chapter 12.4), it follows that

$$P\left(\underset{B\in A_{\epsilon_{n}^{*}}}{\sup}\left|{\hat{\mu }}_{n}\left(B\right)-\mu_{\theta }\left(B\right)\right|>\delta \right)\leq 8{\left(n+1\right)}^{J}\cdot e^{-\frac{n\delta^{2}}{32}},$$

∀n≥0 and ∀ϵ>0. Then considering $a_{n}=\sqrt{\log n/n}$ and $M^{2}/32>J+2$,

$$P\left(\underset{B\in A_{\epsilon_{n}^{*}}}{\sup}\left|{\hat{\mu }}_{n}\left(B\right)-\mu_{\theta }\left(B\right)\right|>M\cdot a_{n}\right)\leq 8\frac{{\left(n+1\right)}^{J}}{n^{M^{2}/32}}\leq \frac{K}{n^{2}}$$

for some K>0, hence $\sum_{n\geq 0}P\left(\frac{1}{a_{n}}\cdot \sup_{B\in A_{\epsilon_{n}^{*}}}\left|{\hat{\mu }}_{n}\left(B\right)-\mu_{\theta }\left(B\right)\right|>M\right)<\infty$. Then from the Borel Cantelli Lemma, $\lim\sup_{n\to \infty }\frac{1}{a_{n}}\cdot \sup_{B\in A_{\epsilon_{n}^{*}}}\left|{\hat{\mu }}_{n}\left(B\right)-\mu_{\theta }\left(B\right)\right|\leq M$$P_{\mu }$-almost surely, which concludes the proof. As $\left(a_{n}\right)$ is o(1), this result implies the almost-sure convergences to zero of $V(\mu_{{\hat{\theta }}_{\epsilon_{n}^{*}}\left(X^{n}\right)},\mu_{\theta })$ as *n* goes to infinity.

Finally, using similar arguments, it is possible to show that $V(\mu_{{\hat{\theta }}_{\epsilon_{n}^{*}}\left(X^{n}\right)},\mu_{\theta })$ is $o(1/n^{\tau })$$P_{\mu }$-almost surely for any τ∈(0,1/2). ☐

### 8.5. Lemma 1

**Proof.**  First note that Θ is contained in a compact set ${⨂}_{i=1}^{k}\left[-L,L\right]\subset R^{k}$, consequently, Θ inherits the finite covering property of a compact set, i.e., ∀ϵ>0, there exists a finite covering $\Theta^{\epsilon }=\left\{\theta_{1}^{\epsilon },.,\theta_{K(\epsilon )}^{\epsilon }\right\}\subset \Theta$ such that,

(50) $$\Theta \subset \underset{\theta \in \Theta }{⋃}B_{\epsilon }\left(\theta \right)=\overset{K(\epsilon )}{\underset{i=1}{⋃}}B_{\epsilon }\left(\theta_{i}^{\epsilon }\right).$$

On the other hand, from the locally uniformly Lipschitz assumption on $I_{F}:\Theta \to F$, there exists r>0 and m>0 such that $V\left(\mu_{\theta },\mu_{\phi }\right)\leq m\left|\left|\theta -\phi \right|\right|$, ∀θ∈Θ, $\forall \phi \in B_{r}\left(\theta \right)$. Then, by considering $\epsilon_{o}<r$, it follows by construction of $\Theta^{\epsilon_{o}}$ that

(51) $$\begin{array}{c} F\subset \overset{K(\epsilon_{o})}{\underset{i=1}{⋃}}I_{F}\left(B_{\epsilon_{o}}\left(\theta_{i}^{\epsilon_{o}}\right)\right)\subset \overset{K(\epsilon_{o})}{\underset{i=1}{⋃}}B_{m\cdot r}^{V}\left(\mu_{\theta_{i}^{\epsilon_{o}}}\right), \end{array}$$

where $B_{\delta }^{V}\left(\mu \right)=\left\{v\in P(X):V(v,\mu )<\delta \right\}$ is the ball centered at μ∈P(X), induced from the total variational distance, and the last inequality stems from the Lipschitz condition. Hence, from (51), ∀ϵ>0 there exists $M\left(\epsilon \right)=K\left(\min\left\{\epsilon /m,r\right\}\right)<\infty$ and $\left\{\mu_{1}^{\epsilon },...,\mu_{M(\epsilon )}^{\epsilon }\right\}\subset P\left(X\right)$, such that $F\subset {⋃}_{i=1}^{M(\epsilon )}B\left(\mu_{\theta_{i}^{\epsilon }},\epsilon \right)$, which proves the result.

For the final part, let (m,r) be the uniform parameters that characterize the Lipschitz condition of $I_{F}\left(\cdot \right)$ (Definition 11). Without loss of generality, let us assume the critical regime where $\frac{\epsilon }{m}<r$, hence from (51) $N_{\epsilon }$ is upper bounded by K(ϵ/m), which is the covering number of Θ. As $\Theta \subset {⨂}_{i=1}^{k}\left[-L,L\right]\subset R^{k}$, we will work with a uniform partition of ${⨂}_{i=1}^{k}\left[-L,L\right]$ to find a bound for K(ϵ/m). Let $\bar{\epsilon }=\frac{\epsilon }{m}$, then inducing a product-type partition, where in each coordinate we have $\left\lceil \frac{L\sqrt{k}}{\bar{\epsilon }}\right\rceil$ uniform length cells, we have the required $\bar{\epsilon }$-covering. The number of prototypes is $O(\frac{{\left(L\sqrt{k}\right)}^{k}}{{\bar{\epsilon }}^{k}})$, which is $O(1/\epsilon^{k})$ as a function of ϵ ($\epsilon =\bar{\epsilon }\cdot m$).

To clarify the constructive nature of the ϵ-covering used to prove this result, an algorithm with the basic steps of the construction of this practical covering is sketched in Appendix C. ☐

### 8.6. Theorem 4

**Proof.**  Let ${\tilde{G}}_{\epsilon }\subset F$ be the ϵ-covering induced from the uniform partition of Θ presented in Lemma 1. From this we can construct the minimum-distance estimate in (42) adopting the Yatracos class of ${\tilde{G}}_{\epsilon }$ (with index set ${\tilde{\Theta }}_{\epsilon }$), i.e., ${\tilde{A}}_{\epsilon }$, which, from (39), yields

(52) $${\tilde{\theta }}_{\epsilon }\left(X^{n}\right)\equiv \arg\min_{\theta_{i}^{\epsilon }\in {\tilde{\Theta }}_{\epsilon }}\underset{B\in {\tilde{A}}_{\epsilon }}{\sup}\left|\mu_{\theta_{i}^{\epsilon }}\left(B\right)-{\hat{\mu }}_{n}\left(B\right)\right|.$$

Considering $\epsilon_{n}=1/\sqrt{n}$, from (45) it follows that

$$\underset{\mu_{\theta }\in F}{\sup}E\left\{V(\mu_{{\tilde{\theta }}_{\epsilon_{n}}\left(X^{n}\right)},\mu_{\theta })\right\}\leq \frac{3}{\sqrt{n}}+\sqrt{\frac{8\log(2\log{\left|{\tilde{G}}_{1/\sqrt{n}}\right|}^{2})}{n}}.$$

The latter upper bound is asymptotically dominated by $\left(\sqrt{\log n/n}\right)$ from the fact that $\log\left|{\tilde{G}}_{1/\sqrt{n}}\right|$ is O(klog(n)) (Lemma 1), which proves the assertions made in (26).

Concerning part (ii), using the arguments presented in the proof of Theorem 3, we can obtain that ∀ϵ>0,

(53) $$\underset{\mu_{\theta }\in F}{\sup}E\left\{V(\mu_{{\tilde{\theta }}_{\epsilon }\left(X^{n}\right)},\mu_{\theta })\right\}\leq 3\epsilon +4\cdot c\sqrt{\frac{J}{n}}.$$

From this point, the proof follows from the arguments of Theorem 3 and the fact that $\log_{2}\left|{\tilde{G}}_{1/\sqrt{n}}\right|$ is $O(k/2\cdot \log_{2}n)$. ☐

## Appendix A. Proof of (22) and (23)

First, we show that the zero-rate skeleton estimate $\Pi \left(\left(\epsilon_{n}\right)\right)=\left\{(f_{n,\epsilon_{n}},\phi_{n,\epsilon_{n}}):n\geq 1\right\}$ proposed in (40) and (41) is also strongly consistent.

**Proposition** **A1.**
*$\Pi \left(\left(\epsilon_{n}^{*}\right)\right)=\left\{(f_{n,\epsilon_{n}^{*}},\phi_{n,\epsilon_{n}^{*}}):n\geq 1\right\}$ is strongly consistent, i.e., for any μ∈F,*

$$\begin{array}{c} \lim_{n\to \infty }V\left(\mu_{{\hat{\theta }}_{\epsilon_{n}^{*}}\left(X^{n}\right)},\mu \right)=0,\;P_{\mu }-almost\;surely. \end{array}$$

**Proof.** Let us consider the skeleton estimate $\mu_{{\hat{\theta }}_{\epsilon_{n}^{*}}\left(X^{n}\right)}$, where the sequence was chosen by the rule $\epsilon_{n}^{*}=\inf\left\{\epsilon >0:\log\left(2N_{\epsilon }^{2}\right)\leq \sqrt{n}\right\}$. Then $\log N_{\epsilon_{n}^{*}}^{2}\leq \left(\sqrt{n}-\log2\right)\leq \sqrt{n}$ for all *n*. From Lemma 3, $V\left(\mu_{{\hat{\theta }}_{\epsilon_{n}^{*}}\left(X^{n}\right)},\mu \right)\leq 3\epsilon_{n}^{*}+4\sup_{B\in A_{\epsilon_{n}^{*}}}\left|{\hat{\mu }}_{n}\left(B\right)-\mu \left(B\right)\right|$. As by construction $\left(\epsilon_{n}^{*}\right)$ is o(1), we just need to concentrate on the estimation error term. Applying Hoeffding’s inequality [44] ∀δ>0,

(A1) $$\begin{array}{cc} P\left(\underset{B\in A_{\epsilon_{n}^{*}}}{\sup}\left|{\hat{\mu }}_{n}\left(B\right)-\mu \left(B\right)\right|>\delta \right) & \leq 2\cdot N_{\epsilon_{n}^{*}}^{2}\cdot e^{-2n\delta^{2}}\leq 2e^{\left(\sqrt{n}/\log e-2n\delta^{2}\right)}, \end{array}$$

where from the Borel-Cantelli lemma [46,47], the estimation error convergences to zero almost-surely. ☐

Finally considering the inequality in (37), we have that $D_{\mu }\left(C^{n,n}|Z^{n}\right)-D_{\mu }^{n}\left(R\right)\leq$$2^{1/p+1}d_{max}\cdot V\left(\mu_{{\hat{\theta }}_{n}\left(Z^{n}\right)},\mu \right)$, ∀μ∈F, which concludes the argument.

## Appendix B. Basic Definitions of Vapnik and Chervonenkis Theory

Let C⊂B(X) be a collection of measurable events, and $x^{n}=\left(x_{1},...,x_{n}\right)$ be a sequence of *n* points in $X^{n}$. Then we define by $S(C,x^{n})$ the number of different sets in

$$\left\{\left\{x_{1},x_{2},\ldots ,x_{n}\right\}\cap B:B\in C\right\},$$

and the shatter coefficient of C by [40,45]

(A2) $$S_{n}\left(C\right)=\underset{x^{n}\in X^{n}}{\sup}S\left(C,x^{n}\right).$$

The shatter coefficient is an indicator of the richness of C to dichotomize a finite sequence of points in the space, where by definition $S_{n}\left(C\right)\leq 2^{n}$.

**Definition** **A1.**
*The first time (in the index n) where $S_{n}\left(C\right)$ is strictly less than $2^{n}$ is called the Vapnik and Chervonenkis (VC) dimension of C [45]. If C has a finite VC dimension then it is called a VC class; otherwise if $S_{n}\left(C\right)=2^{n}$∀n≥1, then the class is said to have an infinite VC-dimension.*

## Appendix C. Pseudo Algorithm to Implement the Practical *ϵ*-Covering Presented in Lemma 1

Under the parametric assumptions of Lemma 1, we recognize four structural parameters that characterize F: *k* the dimension of the Euclidean space that contains Θ, L>0 associated with the assumption that $\Theta \subset {⨂}_{i=1}^{k}\left[-L,L\right]$, and (r,m) the parameters associated with the locally Lipschitz assumption of $I_{F}$. Given these four parameters (k,L,m,r) and ϵ>0, there is a constructive ϵ-covering presented in the proof of Lemma 1 that can be implemented in the following steps:

1. In each of the *k* dimensions of Θ, the interval [−L,L] is partitioned uniformly with sub-intervals of length $2\epsilon /(m\sqrt{k})$. This produces a scalar quantization of [−L,L] with $\left\lceil m\sqrt{k}L/\epsilon \right\rceil$ prototypes per coordinate.
2. A product partition of ${⨂}_{i=1}^{k}\left[-L,L\right]$ is made with the scalar quantizations of the previous step. From the proof of Lemma 1, this is a ϵ/m-covering of Θ with $K={\left\lceil m\sqrt{k}L/\epsilon \right\rceil }^{k}$ prototypes. Let us denote this set by $\left\{\theta_{i},i=1,\ldots ,K\right\}\subset \Theta$.
3. From the proof of Lemma 1, the covering of Θ constructed in the previous step induces an ϵ-covering of F by applying the indexing function $I_{F}$, i.e., by $\left\{I_{F}\left(\theta_{i}\right):i=1,\ldots ,K\right\}.$
